# Application of a Deep Learning Algorithm for Combined Super-Resolution and Partial Fourier Reconstruction Including Time Reduction in T1-Weighted Precontrast and Postcontrast Gradient Echo Imaging of Abdominopelvic MR Imaging

**DOI:** 10.3390/diagnostics12102370

**Published:** 2022-09-29

**Authors:** Daniel Wessling, Judith Herrmann, Saif Afat, Dominik Nickel, Haidara Almansour, Gabriel Keller, Ahmed E. Othman, Andreas S. Brendlin, Sebastian Gassenmaier

**Affiliations:** 1 Department of Diagnostic and Interventional Radiology, University Hospital of Tuebingen, Hoppe-Seyler-Strasse 3, 72076 Tuebingen, Germany; 2 MR Application Predevelopment, Siemens Healthcare GmbH, Allee am Roethelheimpark 2, 91052 Erlangen, Germany; 3 Department of Neuroradiology, University Medical Center, 55131 Mainz, Germany

**Keywords:** MRI, deep learning, abdominal, pelvic

## Abstract

Purpose: The purpose of this study was to test the technical feasibility and the impact on the image quality of a deep learning-based super-resolution reconstruction algorithm in 1.5 T abdominopelvic MR imaging. Methods: 44 patients who underwent abdominopelvic MRI were retrospectively included, of which 4 had to be subsequently excluded. After the acquisition of the conventional volume interpolated breath-hold examination (VIBE_Std_), images underwent postprocessing, using a deep learning-based iterative denoising super-resolution reconstruction algorithm for partial Fourier acquisitions (VIBE_SR_). Image analysis of 40 patients with a mean age of 56 years (range 18–84 years) was performed qualitatively by two radiologists independently using a Likert scale ranging from 1 to 5, where 5 was considered the best rating. Results: Image analysis showed an improvement of image quality, noise, sharpness of the organs and lymph nodes, and sharpness of the intestine for pre- and postcontrast images in VIBE_SR_ compared to VIBE_Std_ (each *p* < 0.001). Lesion detectability was better for VIBE_SR_ (*p* < 0.001), while there were no differences concerning the number of lesions. Average acquisition time was 16 s (±1) for the upper abdomen and 15 s (±1) for the pelvis for VIBE_Std_, and 15 s (±1) for the upper abdomen and 14 s (±1) for the pelvis for VIBE_SR_. Conclusion: This study demonstrated the technical feasibility of a deep learning-based super-resolution algorithm including partial Fourier technique in abdominopelvic MR images and illustrated a significant improvement of image quality, noise, and sharpness while reducing TA.

## 1. Introduction

In the last decades, magnetic resonance imaging (MRI) has become the first modality of choice in the investigation of abdominal and pelvic pathologies such as chronic inflammatory bowel diseases, pathologies of the urogenital tract, and local tumor staging in malignancies [1,2]. Due to the development of faster MRI sequences, the modality even became an alternative to other modalities in certain emergency cases, especially in pregnant women and children [3,4,5].

One of the biggest challenges in abdominopelvic imaging are motion artifacts [6]. Although conventional turbo spin echo (TSE) MRI sequences provide good image quality with a good signal-to-noise ratio (SNR) in abdominopelvic imaging, motion artifacts still represent a major issue, particularly in imaging of the upper abdominal organs [7]. One approach to handle this problem (especially in contrast-enhanced imaging) is gradient echo (GRE)-based MR imaging. GRE sequences use a much shorter repetition time (TR), and therefore allow a significant reduction of the acquisition time (TA) [8,9]. Limitations of GRE imaging are the vulnerability to magnetic field inhomogeneity and the susceptibility to artifacts [10]. Despite these issues, contrast enhanced, three-dimensional, T1-weighted, GRE-based sequences have prevailed in clinical routine abdominopelvic imaging [11].

Nevertheless, for the image acquisition in GRE images, several breath-holds are needed so that good cooperation from the patient during the examination has a significant influence on the image quality. To tackle this problem, several free breathing approaches have been developed to make MR image quality as independent as possible from breathing commands which are, however, not yet established in clinical routine [12,13].

Conventional acceleration techniques, as parallel imaging, allow an acceleration of GRE imaging with the disadvantage of SNR loss proportional to the square root of the acceleration factor [14,15,16,17]. However, in the last decade, deep learning-based imaging has been investigated for automatic image analysis as well as for further acceleration of MRI [18,19,20,21,22]. Deep learning-based sequences have shown the potential to reduce TA while maintaining good image quality. Nevertheless, only a few studies have tested the clinical application of deep learning-based sequences. Therefore, clinical implementation of deep learning-based MRI will require more time and research to gain further insights.

Another interesting deep learning approach that could be implemented more easily is deep learning-based postprocessing. Deep learning-based reconstructions allow a further improvement of image quality compared to compressed sensing and parallel imaging [23,24]. In former studies, we could show the possibility of theoretical acquisition time reduction via postprocessing due to the application of partial Fourier method in GRE imaging of the upper abdomen and the pancreas in particular [25,26]. As these studies showed a significant improvement of image quality on the upper abdominal organs, in this study we want to analyze the super-resolution algorithm on a whole abdominopelvic MRI scan while also focusing on the pelvis and the intestine.

As former studies have primarily shown improvements in noise, our aim is to investigate further image parameters that could be improved by the investigated super-resolution reconstruction algorithm.

Therefore, we are presenting the technical feasibility of a novel super-resolution-based reconstruction technique in abdominopelvic MR imaging including an evaluation of image quality, noise, sharpness of the organs and lymph nodes, sharpness of the intestine, the level of artifacts, and lesion detectability.

In our article, we illustrate the technique and implementation of the applied algorithm in a clinical abdominopelvic MRI protocol including pre- and postcontrast sequences of the pelvis. The results show the impact of the super-resolution algorithm on several image quality parameters. Finally, we discuss the relevance of the novel super-resolution technique and offer a short forecast for possible research opportunities.

## 2. Material and Methods

### 2.1. Study Design

This monocentric, retrospective, single institutional study was approved with a waiver of informed consent by the local institutional review board. The study was conducted following the ethical standards of the Declaration of Helsinki from 1964 and its latest revision in 2013. *n* = 44 patients who received an abdominopelvic MRI examination with a 1.5 T scanner in our radiology department were retrospectively included in the study.

### 2.2. Acquisition Parameters

All MRI examinations were performed in a clinical routine setting using 1.5 T scanners (Aera and Avanto^fit^, Siemens Healthcare, Erlangen, Germany). Patients were examined in a supine position using a 32-channel spine coil and an 18-channel body coil. The standard clinical protocol comprised the following sequences: 1. Axial standard T1w VIBE (VIBE_Std_) precontrast with fat suppression using the Dixon method. 2. Axial standard T1w VIBE postcontrast with fat suppression using the Dixon method in the equilibrium phase approximately three minutes after contrast agent application. 3. Axial T2w BLADE using the periodically rotated overlapping parallel lines with enhanced reconstruction (PROPELLER) technique. 4. Coronal T2w Half Fourier Single-shot Turbo spin-Echo (HASTE). 5. Axial diffusion weighted imaging using two different b-values (0 and 1000 s/mm^2^).

MRI examinations were conducted using a body-weight-adapted intravenous contrast agent injection (0.1 mmol/kg gadobutrol (Gadovist, Bayer Healthcare, Berlin, Germany)) with a flow rate of 1.5 mL/s and followed by a saline flush of 20 mL.

Axial VIBE pre- and postcontrast images were acquired using two composed acquisitions with the following parameters: voxel size of 1.3 × 1.3 × 3 mm^3^, slice thickness of 3 mm, number of slices of 88, a TR of 6.5 milliseconds, echo times (TE) of 2.39/4.77 milliseconds, a flip angle of 10 degrees, a parallel imaging factor of 4, phase partial Fourier 7/8, slice partial Fourier 6/8, and a TA of 13 s.

### 2.3. Deep Learning Super-Resolution Postprocessing

**Figure 1:**
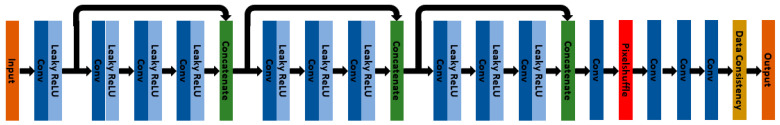
Network architecture used in this work. The input image volume passes through a sequence of ten convolutions (deep blue) followed by leaky rectified unit activations (light blue). Three skip connections using concatenation (green) are inserted. After a subsequent convolution, pixel shuffling is applied for reshaping channels into interleaved voxels for upsampling. Finally, the obtained high-resolution image is filtered by three convolutions, and a data consistency projection is performed in the Fourier domain.

After the acquisition of the conventional VIBE_Std_ sequence used in clinical routine, the corresponding raw data were reprocessed on the MRI scanner using a prototypical reconstruction integrated into the vendor’s processing pipeline that can be triggered manually. The prototypical reconstruction was configured to omit data that are outside of a specified range of phase-encoding steps so that shorter acquisitions could be simulated. Further, after image creation in the pipeline, the intermediate images were fed into a super-resolution network that was trained on input images with a network-specific amount of partial Fourier sampling, resulting in the deep learning-based super-resolution dataset VIBE_SR_. Training data were generated with increased resolution in head and pelvic imaging in volunteers with acquisition times ranging from one to three minutes. The employed network was trained for a slice partial Fourier factor of 0.75 and corresponds to the network used in Ref. [25] from our research group (Figure 1).

### 2.4. Image Analysis

Image analysis was performed using a dedicated workstation (Centricity PACS RA1000; GE Healthcare, Milwaukee, WI, USA). All images were rated qualitatively by two independent radiologists with 2 and 7 years’ experience in MR imaging in a random blinded order and without any access to the patient history or the original radiological report. Image analysis was performed using a Likert-scale ranging from 1 to 5.

All images were rated for overall image quality (1, nondiagnostic; 2, highly reduced image quality; 3, moderate image quality; 4, good image quality; 5, excellent image quality), noise levels (1, nondiagnostic; 2, high noise; 3 moderate noise; 4, little noise; 5, almost no noise), sharpness of organs, lymph nodes, and the intestine (1, nondiagnostic; 2, highly reduced sharpness; 3, moderate sharpness; 4, high sharpness; 5, excellent sharpness), artifacts (1, nondiagnostic; 2, high level of artifacts; 3, moderate level of artifacts; 4, low level of artifacts; 5, almost no artifacts) and lesion detectability (1, nondiagnostic; 2, lesion barely detectably; 3, moderate lesion detectability; 4, good lesion detectability; 5, excellent lesion detectability). The rating was performed using a Likert-scale ranging from 1 to 5, whereas reading scores ≥ 3 were considered as sufficient for clinical use.

In all examinations, the lesion with the largest diameter was measured by both readers.

### 2.5. Statistical Analysis

Statistical analysis was performed using dedicated statistical programs MedCalc Statistical Software version 18.10 (MedCalc Software bvba, Ostend, Belgium) and jmp (jmp15, MP^®^, Version 15 SAS Institute Inc., Cary, NC, USA, 1989–2019). Both parametric and nonparametric values are shown using median and interquartile range (IQR). Comparison of the ordinal, qualitative data was performed using the Wilcoxon-signed-rank test. The inter-rater reliability was tested using linearly weighted Cohens κ, whereas values ≤ 0 indicate no agreement, 0.01–0.20 were rated as none to a slight agreement, 0.21–0.40 as fair agreement, 0.41–0.60 as moderate agreement, 0.61–0.80 as substantial agreement, and 0.81–1.00 as almost perfect agreement [27]. Wilcoxon-singed-rank test was used to compare the numeric data. Inter-rater agreement was tested using the ICC-correlation coefficient with values < 0.5 indicating a poor agreement, 0.5–0.75 indicating a moderate agreement, 0.75–0.90 indicating a good agreement, and 0.90–1.0 indicating a perfect agreement [28].

## 3. Results

### 3.1. Patient Cohort

*n* = 4 of the *n* = 44 patients retrospectively included patients had to be subsequently excluded. In two patients, the abdominopelvic MRI was conducted as part of an MR imaging with a different protocol: *n* = 1 patient received a whole-body MRI, and in *n* = 1 patient, the imaging was performed during an MR-angiography of the aorta. As the imaging protocol and the acquired sequences differed from our study protocol, these patients had to be excluded. In *n* = 2 cases, the sequences were incomplete, so the patients were excluded from the study.

Of *n* = 40 patients, *n* = 36 patients underwent the MRI examination for a follow-up of a histopathological proven malignancy, while *n* = 4 patients had unclear symptoms that needed to be clarified by MRI. A detailed subdivision of the reasons for the examination can be found in Table 1. The median age was 56 ± 17 years with a range from 18–84 years; 24 patients were female, and 16 patients were male.

### 3.2. Image Analysis

The test for inter-rater reliability showed a substantial agreement (κ = 0.733). Thus, we decided to discuss only the results of the first reader. The detailed results of both readers are listed in Table 2 and Table 3.

### 3.3. Qualitative Results of the Precontrast Images

**Figure 2:**
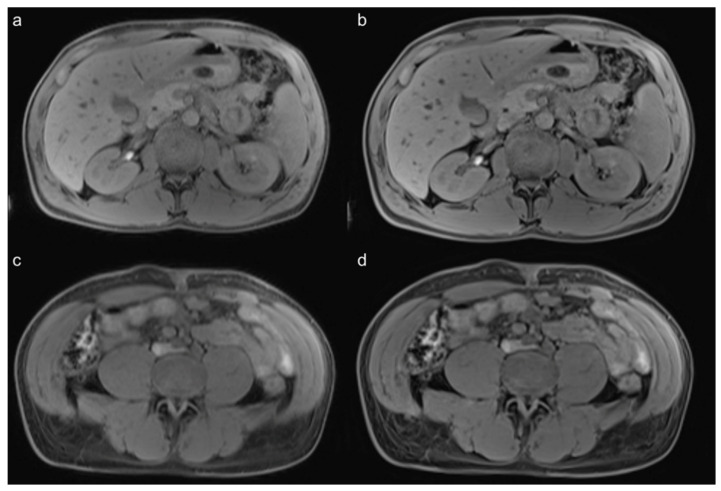
Precontrast images of a 28-year-old male patient with unclear elevation of the AFP-levels in his blood samples. In the MR images which were made to exclude an extragonadal tumor, the noise, organ sharpness, and sharpness of the intestine are better in the VIBE_SR_ (**b**,**d**) than in the VIBE_Std_ (**a**,**c**).

Image quality, noise, sharpness of the organs and lymph nodes, sharpness of the intestine, and artifacts were significantly better for VIBE_SR_ (each *p* < 0.001). The most significant differences between VIBE_Std_ and VIBE_SR_ were found concerning noise, sharpness of the organs and the lymph nodes, and sharpness of the intestine that were all rated with a median of 4 (IQR 3–4) for VIBE_Std_ and with a median of 5 (IQR 5–5) for VIBE_SR_. An example of the improvement of quality in VIBE_SR_ is shown in Figure 2.

### 3.4. Qualitative Results of the Postcontrast Images

**Figure 3:**
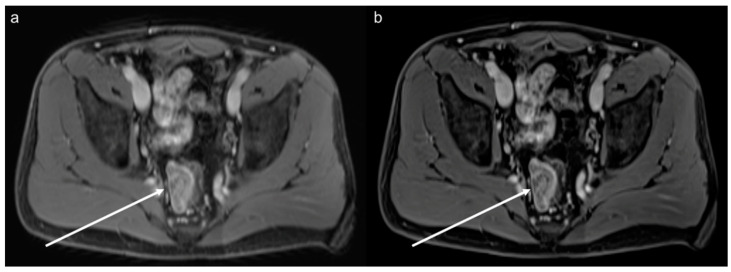
Images of a 45-year-old male patient who underwent MRI for staging because of a newly diagnosed melanoma. As an incidental finding, the images show a suspicious contrast uptake in a lesion of the right rectum (arrow) which proved to be rectal carcinoma. The VIBE_SR_ images (**b**) show a better SNR and sharpness, thus offering more thorough information about the local extension and possible lymph node metastases than the VIBE_Std_ (**a**).

**Figure 4:**
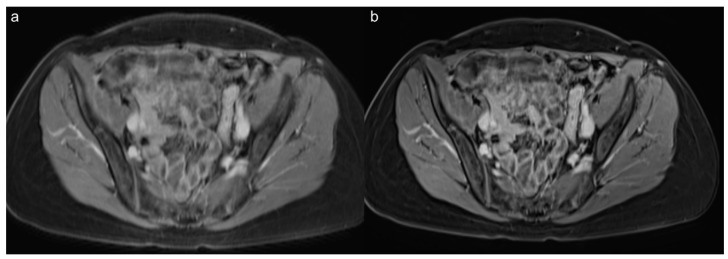
Follow-up abdominopelvic MRI in a 51-year-old male patient who had undergone Whipple surgery because of neuroendocrine neoplasia of the papilla VATERI. The postcontrast VIBE_SR_ images (**b**) show a distinctively better SNR and sharpness of the lymph nodes and the intestine than the VIBE_Std_ postcontrast VIBE images (**a**).

**Figure 5:**
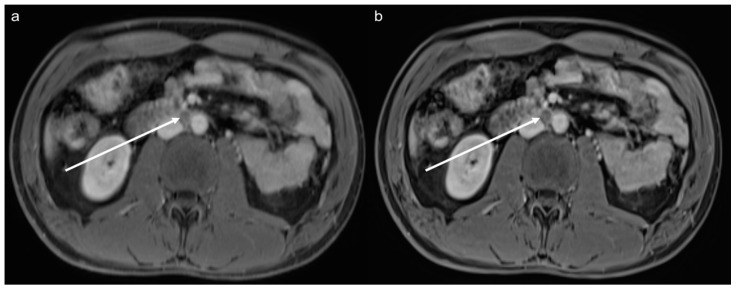
Staging of a 29-year-old male patient after surgical resection of an embryonal cell carcinoma of the testicle. The images show a hypointense retroperitoneal lymph node metastasis (arrow). Due to a higher SNR and a better sharpness of the lymph nodes, organs, and the intestine, the metastasis and surrounding structures can be better differentiated and assessed in the postcontrast VIBE_SR_ image (**b**) than in the VIBE_Std_ image (**a**).

Corresponding to the qualitative results of the precontrast images, similar differences were also found in the postcontrast images. The rating for the VIBE_SR_ was significantly better in terms of image quality, noise, sharpness of the organs and lymph nodes, and sharpness of the intestine (each *p* < 0.001). The biggest differences were found regarding image quality, noise, and sharpness of the intestine. Image quality was rated with a median of 4 (IQR 3–4) for VIBE_Std_ and with a median of 5 (IQR 5–5) for VIBE_SR_. Median for noise was 4 (IQR 3.5–4) for VIBE_Std_ and 5 (IQR 5–5) for VIBE_SR_, and median for sharpness of the intestine was 4 (IQR 3–4) for VIBE_Std_ and 5 (IQR 5–5) for VIBE_SR_. The image quality improvement in VIBE_SR_ is displayed in Figure 3, Figure 4 and Figure 5.

### 3.5. Lesion Assessment

In 31 of 40 MRI scans, a lesion could be detected. There was no difference between the number of detected lesions in both readers. The evaluation showed no statistically significant differences regarding lesion size between VIBE_Std_ (11 mm (IQR 7–25 mm)) and VIBE_SR_ (12 mm (IQR 7–26 mm)) for reader 1 (*p* = 0.173) and between VIBE_Std_ (11 mm (IQR 7–25 mm)) and VIBE_SR_ (12 mm (IQR 7–26 mm)) for reader 2 (*p* = 0.625) for neither the precontrast nor postcontrast images. Inter-rater reliability, tested with ICC, was 0.998 for the VIBE_Std_ and 0.999 for VIBE_SR_. The detailed results of the lesion assessment are listed in Table 4 and Table 5.

### 3.6. Acquisition Time

Average acquisition time was 16 sec (±1) for the upper abdomen and 15 sec (±1) for the pelvis for VIBE_Std_ and 15 sec (±1) for the upper abdomen and 14 sec (±1) for the pelvis for VIBE_SR_.

## 4. Discussion

This study investigated the technical feasibility and clinical applicability of a novel deep learning-based super-resolution image technique fitted to partial Fourier acquisitions of T1-weighted precontrast and postcontrast abdominopelvic GRE imaging. The study shows an improvement of overall image quality, noise, sharpness of the organs and lymph nodes, sharpness of the intestine, artifacts, and lesion detectability, while reducing TA.

GRE imaging such as the VIBE_Std_ is a widely used and approved imaging technique in abdominopelvic MRI. Nevertheless, these sequences show a high susceptibility to artifacts as being very sensitive to magnetic field inhomogeneities [8]. Another problem of GRE-based imaging is the necessity of several breath-holds, particularly in abdominopelvic MRI, which can be a limiting factor for uncooperative patients, elderly patients, and patients with respiratory preconditions. Therefore, much effort has been made to approach this aspect via free-breathing GRE sequences, including a free-breathing 3D VIBE sequence that can improve lesion conspicuity and lower the artifacts in MR images [29,30]. However, free-breathing sequences often lead to an extension of TA. Thus, these sequences may be an advantage for patients who cannot perform breath-holds due to health restrictions. Nevertheless, these sequences are still no adequate solution for patients who are unable to lie quietly for a longer time, for uncooperative patients, or for dynamic contrast-enhanced imaging. In parallel imaging, the time required for the breath-holds and TA can be reduced by subsampling the k-space at the expense of a lower SNR. Our study could show that the used super-resolution algorithm can significantly improve image quality and sharpness while reducing TA via partial Fourier technique.

Therefore, the presented deep learning-based super-resolution postprocessing approach might be a solution to the higher noise levels in parallel imaging. In contrast to compressed sensing, which proved to be very useful in reducing time for breath-holds in MRI, no advanced computational systems are necessary for this kind of postprocessing. The postprocessing can be completed at the conventionally used MRI scanner directly after acquisition. The advantage is, also compared to parallel imaging and compressed sensing, that the standard examination protocol does not have to be changed. Another benefit consists of the retrospective omission of acquired data. On the one hand, the application in the clinical routine would therefore not imply any change of workflows for the medical staff. On the other hand, the original data are always available for standard reconstruction, and no data are lost. The partial omission of data via this super-resolution algorithm that is mimicking more aggressive partial Fourier factors also leads to a reduction of breath-hold time and motion artifacts associated with breathing which naturally occur more often in longer breath-holds. Especially when assessing the bowel and the pelvis, motion artifacts, caused for example by the bowel motility, can also be a major issue [31,32]. Our study has shown a significant improvement of the intestinal sharpness and the level of artifacts by the implementation of a new super-resolution algorithm that might facilitate the evaluation of the intestine and adjacent structures, particularly in the small pelvis.

Former investigated reconstruction algorithms in abdominal or pelvic MRI mainly improved noise and TA. The super-resolution algorithm additionally improves overall image quality, sharpness, and lesion detectability, and, therefore, allows a further improvement of MR imaging [24]. Published results of our recent studies testing the super-resolution algorithm in MRI of the upper abdomen und the pancreas could be confirmed by this study [25,26]. In addition, we could show the technical feasibility and image quality improvement in a whole abdominopelvic MRI scan, which is used more frequently in patients with unclear findings and as staging modality in patients with malignant tumors.

While many of the current studies state that image quality of novel sequences or reconstruction algorithms was slightly impaired or similar, the super-resolution algorithm allowed a significant improvement in almost all image parameters and, therefore, proved to be superior to VIBE_Std_ [33,34]. Especially in pelvic imaging, there are only a few studies investigating accelerated deep learning-based MRI reconstruction algorithms or sequences that also focus mainly on noise reduction [35]. To the best of our knowledge, this is the first study investigating the use of deep learning-based algorithms in postcontrast MR sequences of the pelvis. Furthermore, this study also focused on the assessability of the intestine and could show an improvement of sharpness by implementing VIBE_SR_.

The artifact-free and high-resolution imaging is of enormous importance, especially in young adults and children, where MRI can be used as a radiation-free alternative, for example, in the staging of soft tissue tumors or in the clarification of unclear conditions. As GRE sequences are very sensitive to magnetic field inhomogeneities, VIBE_SR_ could be a technically feasible solution to this problem and reduce the noise levels and thereby improve image quality and sharpness. Furthermore, the reduction of TA and breath-hold time, independently of the improved image quality, could improve the acceptance of MRI examinations in older, multimorbid patients and children who often have difficulties with long-lasting breath-holds. As the algorithm significantly improves the image quality of the intestine and the pelvic organs, deep learning could facilitate the assessment of the intestine in MRI despite bowel movements. In pelvic imaging in particular, radiologists are often faced with complex situations due to the proximity of the pelvic organs. Thus, a high degree of image resolution is necessary to adequately distinguish the respective structures from one another. Due to an improvement in sharpness, small pelvic structures and pathologies could be identified and assessed more accurately. As especially in pelvic imaging there are still just a few studies investigating the utility of deep learning in MRI, further studies will be necessary to confirm our findings. This could also include the implementation of deep learning algorithms in MRI enterography and MRI defecography where deep learning-based algorithms might be an alternative to the use of currently necessary medications [36].

### Limitations

Some limitations of the study have to be considered. Firstly, we did not further evaluate the MRI findings regarding benign or malignant criteria, so no conclusions on the impact of the specificity can be drawn. Secondly, all images were evaluated retrospectively. Further investigations on the possibility of a further time reduction using different settings of data omission via application of more aggressive partial Fourier factors and the impact on the specificity will have to be performed. In addition, the study was performed on a patient cohort of 40 patients with a variety of different underlying diseases. Further research will be needed to confirm the results of this and recently published studies on the practicality of this treatment for individual underlying conditions.

In conclusion, this study illustrated the technical feasibility of deep learning-based super-resolution adapted to partial Fourier acquisition in 1.5 T T1-weighted GRE imaging in abdominopelvic imaging and showed a significant improvement of the image quality, noise, sharpness, level of artifacts, and lesion detectability, while reducing TA.

## Figures and Tables

**Table 1:** Patient characteristics.

Patients (Male/Female), *n*	40 (16/24)
Age, mean ± SD (range), y	total: 56 ± 17 (18–84)
	male: 57 ± 18 (29–84)
	female: 55 ± 16 (18–79)
Diagnosis, *n*	Neuroendocrine neoplasia, 12
	Sarcoma, 6
	GIST, 4
	Melanoma, 4
	Further diagnostic clarification of unclear symptoms, 4
	Urothelial carcinoma, 3
	Testicular cancer, 2
	Breast cancer, 2
	Ovarian/fallopian tube malignancy, 2
	Lymphoma, 1

*n* = number; SD = standard deviation; y, year.

**Table 2:** Results of the precontrast image analysis.

Precontrast Images	Reader 1			Reader 2		
	VIBE_Std_ Median (IQR)	VIBE_SR_ Median (IQR)	*p*-Value	VIBE_Std_ Median (IQR)	VIBE_SR_ Median (IQR)	*p*-Value
**Image Quality parameters**						
IQ	4 (4–4)	5 (5–5)	<0.001	4 (4–4.5)	5 (5–5)	<0.001
Noise	4 (3–4)	5 (5–5)	<0.001	4 (3–4)	5 (4–5)	<0.001
Sharpness organs and lymph nodes	4 (3–4)	5 (5–5)	<0.001	4 (4–4)	5 (5–5)	<0.001
Sharpness intestine	4 (3–4)	5 (5–5)	<0.001	4 (3–4)	5 (5–5)	<0.001
Artifacts	4 (4–4)	5 (4–5)	<0.001	4 (4–4)	5 (4–5)	<0.001

IQ = image quality; DC = diagnostic confidence; IQR = interquartile range.

**Table 3:** Results image analysis postcontrast images.

Postcontrast Images	Reader 1			Reader 2		
	VIBE_Std_ Median (IQR)	VIBE_SR_ Median (IQR)	*p*-Value	VIBE_Std_ Median (IQR)	VIBE_SR_ Median (IQR)	*p*-Value
**Image Quality parameters**						
IQ	4 (3–4)	5 (5–5)	<0.001	4 (4–5)	5 (5–5)	<0.001
Noise	4 (3.5–4)	5 (5–5)	<0.001	4 (4–4)	5 (5–5)	<0.001
Sharpness organs and lymph nodes	4 (4–4.5)	5 (5–5)	<0.001	4 (4–5)	5 (4–5)	<0.001
Sharpness intestine	4 (3–4)	5 (5–5)	<0.001	4 (3–4)	4 (4–5)	<0.001
Artifacts	4 (4–4)	5 (4–5)	<0.001	4 (4–4)	5 (4–5)	<0.001

IQ = image quality; DC = diagnostic confidence; IQR = interquartile range.

**Table 4:** Lesion assessment precontrast images.

Precontrast Images	Reader 1			Reader 2		
	VIBE_Std_ Median (IQR)	VIBE_SR_ Median (IQR)	*p*-Value	VIBE_Std_ Median (IQR)	VIBE_SR_ Median (IQR)	*p*-Value
Lesion size (mm)	11 (7–25)	12 (7–26)	0.173	11 (7–26)	12 (7–26)	0.625
Lesion detectability	4 (4–5)	5 (4–5)	<0.001	4 (4–5)	5 (5–5)	0.003

IQR, Interquartile Range.

**Table 5:** Lesion assessment postcontrast images.

Postcontrast Images	Reader 1			Reader 2		
	VIBE_Std_ Median (IQR)	VIBE_SR_ Median (IQR)	*p*-Value	VIBE_Std_ Median (IQR)	VIBE_SR_ Median (IQR)	*p*-Value
Lesion size (mm)	11 (7–25)	12 (7–26)	0.173	11 (7–26)	12 (7–26)	0.625
Lesion detectability	4 (4–5)	5 (5–5)	<0.001	4 (4–5)	5 (5–5)	<0.001

IQR, interquartile range.

## Data Availability

The data presented in this study are available on request from the corresponding author. The data are not publicly available due to data privacy restrictions.
